# Circular RNA-Drug Association Prediction Based on Multi-Scale Convolutional Neural Networks and Adversarial Autoencoders

**DOI:** 10.3390/ijms26041509

**Published:** 2025-02-11

**Authors:** Yao Wang, Xiujuan Lei, Yuli Chen, Ling Guo, Fang-Xiang Wu

**Affiliations:** 1 School of Computer Science, Shaanxi Normal University, Xi’an 710119, China; wangyao_@snnu.edu.cn (Y.W.); chenyuli@snnu.edu.cn (Y.C.); 2 College of Life Sciences, Shaanxi Normal University, Xi’an 710119, China; 3 Division of Biomedical Engineering, Department of Mechanical Engineering and Department of Computer Science, University of Saskatchewan, 57 Campus Drive, Saskatoon, SK S7N 5A9, Canada

**Keywords:** circular RNA-drug association prediction, multi-scale convolutional neural network, adversarial autoencoder

## Abstract

The prediction of circular RNA (circRNA)-drug associations plays a crucial role in understanding disease mechanisms and identifying potential therapeutic targets. Traditional methods often struggle to cope with the complexity of heterogeneous networks and the high dimensionality of biological data. In this study, we propose a circRNA-drug association prediction method based on multi-scale convolutional neural networks (MSCNN) and adversarial autoencoders, named AAECDA. First, we construct a feature network by integrating circRNA sequence similarity, drug structure similarity, and known circRNA-drug associations. Then, unlike conventional convolutional neural networks, we employ MSCNN to extract hierarchical features from this integrated network. Subsequently, adversarial characteristics are introduced to further refine these features through an adversarial autoencoder, obtaining low-dimensional representations. Finally, the learned representations are fed into a deep neural network to predict novel circRNA-drug associations. Experiments show that AAECDA outperforms various baseline methods in predicting circRNA-drug associations. Additionally, case studies demonstrate that our model is applicable in practical related tasks.

## 1. Introduction

Circular RNAs (circRNAs) represent a class of non-coding RNA molecules characterized by a closed-loop structure, present across various organisms such as animals, plants, and microorganisms. These circRNAs are predominantly formed from precursor mRNA (pre-mRNA) through a process known as back-splicing, where the upstream splice donor connects with the downstream splice acceptor, resulting in a circular configuration. This distinctive circular formation makes circRNAs more resistant to degradation by conventional RNA exonucleases, thereby enhancing their stability within the cell [1].

CircRNAs have been demonstrated to be involved in numerous biological processes, with their functions encompassing miRNA sponging, transcription regulation, protein transport, and enhancement of protein-protein interactions [2]. As miRNA sponges, circRNAs can attach to miRNAs, preventing them from interacting with their intended gene targets, thereby influencing gene expression [3]. This capability is often described as the “miRNA sponge” effect. For instance, in bladder cancer, certain circRNAs have been observed to act as miRNA sponges. CircRNAs are also involved in regulating transcription. Recent studies have revealed that circRNAs derived from the *insulin gene* interact with the RNA-binding protein TDP-43, playing a crucial role in the regulation of genes linked to insulin secretion [4]. CircRNAs also play a role in modulating protein function. For instance, *Circ-Amotl1* contributes to wound healing by interacting with *Dnmt3a* and *miR-17*, binding to *STAT3*, and *facilitating* its transport into the nucleus, where it upregulates the expression of fibronectin [5].

In the 1970s, circRNAs were first observed, but due to technological limitations and a lack of understanding of their function, they did not attract widespread attention. In 2012, Salzman et al. [6] first proposed that pre-mRNA could form circular RNA through back-splicing. These circRNAs were found in various *human* cell types. Jeck and colleagues identified over 25,000 specific types of RNA in human fibroblasts [7], thus uncovering the mechanism of circRNA formation. Since then, research on circRNAs has quickly gained significant attention.

Validating the relationships between circRNAs and drugs using conventional biomedical techniques can be both time-intensive and costly. Consequently, the demand for developing computational methods that can accurately and efficiently predict circRNA-drug associations is increasing. The Graph Attention Network (GAT) is a deep learning model designed for graph data, which merges the advantages of Graph Neural Networks (GNNs) with attention mechanisms. By incorporating attention mechanisms to handle graph-structured data, GAT enables the network to prioritize significant neighboring nodes, thereby enhancing the extraction of features from the graph. This model has been widely utilized in predicting circRNA-drug associations as well as in other association prediction tasks [8]. Deng et al. [9] proposed GATECDA, a computational framework utilizing a graph attention autoencoder, for predicting associations between circRNA and drug sensitivity. GATECDA employs a graph attention autoencoder (GATE) to produce compact representations of circRNAs and drugs, preserving critical information from sparse, high-dimensional features while seamlessly incorporating data from neighboring nodes. Additionally, Yang et al. [10] introduced an innovative method called MNGACDA to predict potential circRNA-drug sensitivity associations, aiming to facilitate deeper biomedical research. MNGACDA builds a multimodal network by combining multiple information sources related to circRNAs and drugs. It then utilizes node-level attention-based graph autoencoders to extract low-dimensional embeddings of circRNAs and drugs from this integrated network. In the final step, the model applies an inner-product decoder to compute association scores between circRNAs and drug sensitivity, using the generated embeddings as input. Similarly, Li et al. [11] developed DGATCCDA, a deep learning-driven computational method focused on detecting circRNA-drug sensitivity associations. DGATCCDA begins by constructing a multimodal network using the original feature data of circRNAs and drugs. Next, a DeepWalk-based graph attention network is employed to thoroughly capture feature information from the multimodal network, producing node embeddings. The multimodal network features are then merged via layer attention, followed by applying an inner-product technique to create an association matrix between circRNAs and drugs for prediction.

Moreover, other Graph Neural Network (GNN) approaches, such as Graph Convolutional Networks (GCNs) and autoencoders [12], have also proven effective in addressing the circRNA association prediction challenge [13,14,15]. Specifically, Lei et al. [16]. proposed a method related to the denoising autoencoder and applied it to the prediction of microbe-disease associations. Moreover, the method proposed by Guo et al. [17]. based on an Encoding–Decoding Framework Based on CNN for predicting circRNA–RBP associations has achieved remarkable results. Liu et al. [18] developed a computational framework named GraphCDD to predict associations between circRNAs and drug resistance. By employing multimodal GNNs, GraphCDD integrates various data types to generate effective representations of circRNAs, diseases, and drugs, thereby constructing a predictive model. Lu et al. [19] developed a new method, DHANMKF, to predict possible circRNA-drug sensitivity interactions, with the goal of advancing biomedical validation efforts. DHANMKF constructs a multimodal network by incorporating multiple information sources from circRNAs and drugs. The model then utilizes a dual-type, multi-relational heterogeneous graph to capture detailed intra-type and inter-type node features, which are subsequently enhanced through an attention-based encoder with a hierarchical design. The method integrates these intra-type and inter-type embeddings using a multi-core fusion technique. As the last step, the method employs Dual Laplacian Regularization Least Squares (DLapRLS) to predict potential associations between circRNAs and drug sensitivity within both the circRNA and drug spaces. Li et al. [20] proposed the MNCLCDA computational framework to identify potential circRNA-drug sensitivity associations, with the goal of contributing to medical research advancements. By utilizing drug structural information, circRNA gene sequences, and GIP kernel data, MNCLCDA computes the similarity between a given drug and circRNA. In order to mitigate noise within the similarity data, a preprocessing technique utilizing random walk with restart is implemented, allowing for the efficient extraction of key features from the similarity network. Finally, potential associations are predicted using kernel matrices in the respective feature spaces. The existing links between circRNAs and drug sensitivity are not fully understood, and numerous associations remain unidentified. Consequently, there is a pressing demand for more accurate computational approaches to predict trustworthy circRNA-drug sensitivity relationships.

In this study, we introduce a novel method named AAECDA for predicting circRNA-drug associations aimed at enhancing biomedical screening and validation processes. Our approach integrates circRNA-drug similarity with adversarial autoencoders. At the outset, we compute the combined similarity between circRNAs and drugs by integrating drug structural data with circRNA host gene sequence information, utilizing Gaussian kernel similarity for the calculation. Subsequently, adversarial autoencoders are utilized to learn low-dimensional latent representations from the feature network. Finally, a deep neural network is employed to predict potential circRNA-drug associations. Experimental results across four datasets indicate that AAECDA outperforms existing baseline methods. Additionally, case studies reveal that our model is practically applicable in real-world scenarios.

## 2. Results and Discussion

### 2.1. Experiment Settings and Evaluation Metrics

We applied five (ten) fold cross-validation to assess the model’s effectiveness in predicting circRNA-drug associations. At the outset, the known circRNA-drug associations were considered as positive samples. To create a balanced dataset, a random selection of unobserved circRNA-drug associations was used as negative samples. Both the positive and negative samples were then split equally into five parts. During each round of validation, four parts were used as the training set, while the remaining part served as the test set. The model’s overall performance was then evaluated by calculating the average performance across these five versions. Furthermore, we compared the model’s performance with that of other baseline methods to comprehensively evaluate its strengths and weaknesses.

To assess the performance of the circRNA-drug association prediction model, we utilized seven key metrics: the area under the ROC curve (AUC), the area under the precision-recall curve (AUPR), *F*1 *score*, accuracy, specificity, precision, and recall. These metrics are mathematically defined in Equations (1)–(4). The AUC metric evaluates the model’s capability to accurately distinguish between positive and negative samples. AUC is particularly advantageous because it performs consistently well across various sample distributions, offering high robustness and stability. This helps to minimize performance variability caused by differences in test sets, thereby enabling more precise model evaluations. In binary classification tasks, precision and recall are commonly used to measure a model’s effectiveness. The *F*1 *score*, which is the weighted average of precision and recall, provides a more holistic view of the model’s overall performance. The ROC curve is constructed by plotting the true positive rate (*TPR*) against the false positive rate (*FPR*) across multiple thresholds, providing a comprehensive evaluation of the model’s performance. Similarly, the precision-recall (PR) curve is plotted based on precision and recall values at different thresholds, illustrating the relationship between these two metrics in the prediction task. A higher AUC and AUPR indicate better model performance.

(1) $$TPR=\frac{TP}{TP+FN},\;FPR=\frac{FP}{TN+FP}$$

(2) $$\mathrm{Precison}=\frac{TP}{TP+FP},\;Recall=\frac{TP}{TP+FN}$$

(3) $$\mathrm{Specificity}=\frac{TN}{TN+FP}$$

(4) F1-Score=2×Precision×RecallPrecision+Recall

**Figure 1:**
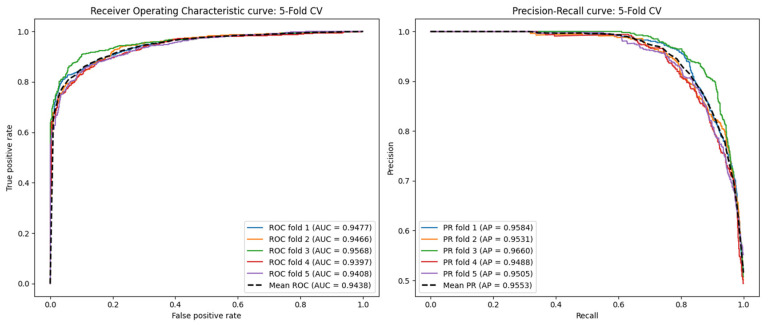
ROC curves and PR curves in fivefold cross-validation.

**Figure 2:**
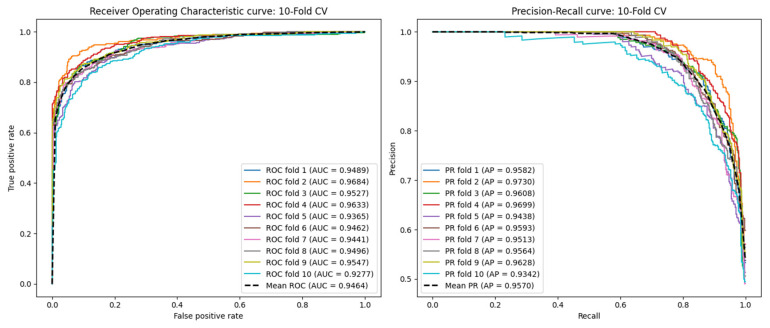
ROC curves and PR curves in tenfold cross-validation.

The ROC and PR curves for the 5-CV scenario are depicted in Figure 1. In this scenario, AAECDA achieves an average AUC of 0.9438 and an average AP of 0.9553. Table 1 presents additional performance metrics, where the average values for *F*1 *score*, accuracy, recall, specificity, and precision are 0.8305, 0.8480, 0.8534, 0.8785, and 0.9302, respectively. For the 10-CV scenario, as shown in Figure 2, AAECDA attains an average AUC of 0.9464 and an average AP of 0.9570. The corresponding metrics, listed in Table 2, show average values of 0.8410 for *F*1 *score*, 0.8407 for accuracy, 0.8437 for recall, 0.8881 for specificity, and 0.9383 for precision.

### 2.2. Performance Comparison with Other Methods Under 5-CV and 10-CV Experiments

Based on available knowledge, existing computational approaches for predicting circRNA-drug interactions remain limited. To assess the predictive capability of AAECDA, we conducted a comparative analysis against five leading models in the field: GATECDA [9], MNGACDA [10], MNCLCDA [20], LAGCN [21], and MKGCN [22] under the same experimental settings. Additionally, the hyperparameters used in the experiments were set according to the recommendations in the authors’ studies. Notably, LAGCN and MKGCN are established models commonly utilized in various bioinformatics prediction tasks, including drug-disease association prediction. Below is a brief overview of each model.

- GATECDA: This computational model employs a graph attention autoencoder and DNN to predict associations between circRNAs and drugs.
- MNGACDA: This model utilizes a node-level attention-based graph autoencoder to extract feature representations and employs an inner-product decoder to predict associations.
- MNCLCDA: This model applies a random walk with a restart method to preprocess the similarity network and capture features, followed by using a mixed-neighborhood graph convolutional network to acquire node neighborhood information.
- MKGCN: This method predicts microbe-drug associations by integrating multiple data sources and applying dual Laplacian regularized fewest squares on multiple kernel matrices.
- LAGCN: The model constructs a heterogeneous network, applies graph convolution to obtain the weights of each layer’s embedding, and then predicts disease-drug associations.

In the 5-CV experiments, as shown in Table 1, the average AUC of AAECDA was 0.9438, which was 5.92% higher than GATECDA, 2.99% higher than MNGACDA, 7.74% higher than MKGCN, and 7.96% higher than LAGCN. The AUPR results are shown in Figure 2. The average AUPR score of AAECDA was 0.9553, which was 6.25% higher than GATECDA, 3.44% higher than MNGACDA, 8.91% higher than MKGCN, and 8.15% higher than LAGCN. Additionally, other performance metrics are shown in Table 1.

In the 10-CV experiments, as shown in Table 2, the average AUC of AAECDA was 0.9464, which was 5.46% higher than GATECDA, 2.82% higher than MNGACDA, 7.81% higher than MKGCN, and 7.54% higher than LAGCN. The AUPR results are shown in Figure 2. The average AUPR score of AAECDA was 0.9570, which was 5.55% higher than GATECDA, 3.21% higher than MNGACDA, 8.01% higher than MKGCN, and 7.52% higher than LAGCN. Additionally, other performance metrics are shown in Table 2.

**Figure 3:**
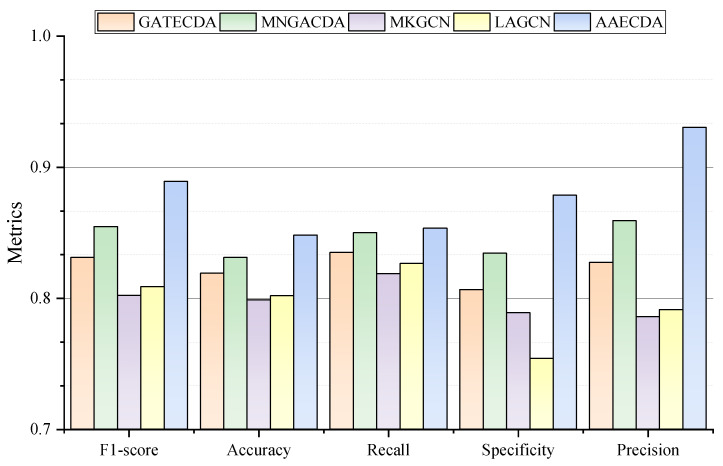
Performance comparison of AAECDA and baseline methods.

Furthermore, as illustrated in Figure 3, the model demonstrates exceptional overall performance. In conclusion, the experiments outlined above confirm that AAECDA is a highly effective computational model for predicting circRNA-drug associations.

### 2.3. Parameter Sensitivity Analysis

The model’s parameters can greatly affect its predictive performance. Therefore, we conducted experiments using 5-fold cross-validation (5-CV) to analyze the sensitivity of some key parameters, including (1) the training ratio $\frac{\lambda_{1}}{\lambda_{2}}$ of the encoder and discriminator, (2) the number of MSCNN convolutional layers *c*.

**Figure 4:**
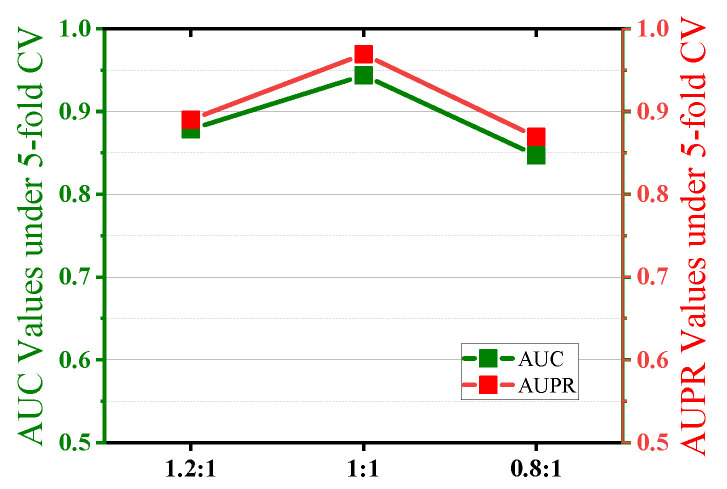
Effect of encoder and discriminator training ratio.

In the AAECDA, the training ratio between the encoder and discriminator is a critical parameter that determines the frequency at which the encoder and discriminator are trained during the adversarial process, directly affecting the model’s balance and final performance. To investigate the impact of different training ratios on model performance, this study selected three training ratios for experimental analysis: 1:1, 1.2:1, and 0.8:1. A ratio of 1:1 means the encoder and discriminator are trained alternately, maintaining a balanced adversarial learning pace. A ratio of 1.2:1 means the encoder is trained 1.2 times before the discriminator is trained once, relatively increasing the training frequency of the encoder. A ratio of 0.8:1 means the encoder is trained 0.8 times before the discriminator is trained once, relatively increasing the training frequency of the discriminator. The experimental results, as shown in Figure 4, indicate that different training ratios have significant effects on metrics such as AUC and AUPR. The specific performance under each training ratio is as follows:

At a ratio of 1:1, the AUC reaches 0.9438, showing the best performance, indicating that the adversarial balance between the encoder and discriminator is ideal at this ratio. At a ratio of 1.2:1, the AUC is 0.8986, decreasing by approximately 4.8% compared to the baseline ratio, indicating a slight decline in performance. This suggests that when the encoder’s training frequency increases, the discriminator’s learning ability is weakened, disrupting the adversarial balance and affecting overall model performance. At a ratio of 0.8:1, the AUC is 0.8769, showing a further decrease of approximately 7.1% compared to the baseline ratio and a further decline compared to 1.2:1. This indicates that while the increased training frequency of the discriminator enhances its discriminative ability, it also suppresses the learning of the encoder, leading to a decrease in the quality of generated features and overall model performance. The experimental findings indicate that when the training ratio is set to 1:1, the training frequencies of the encoder and discriminator remain balanced, providing the most stable and superior adversarial balance during model training.

**Figure 5:**
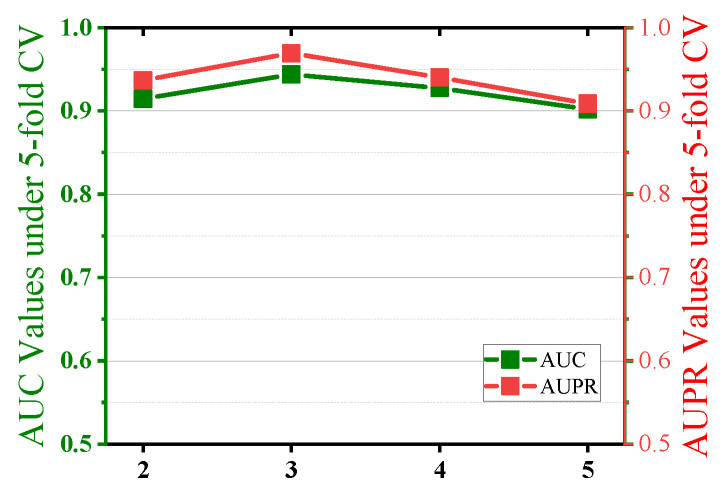
Effect of the number of convolution layers.

Next, the number of convolutional layers c in the multi-scale convolutional neural networks (MSCNN)affects the model’s ability to capture features at different scales. In this study, we set the number of convolutional layers *c* to {2, 3, 4, 5} and observed its impact on model performance. As shown in Figure 5, when the number of convolutional layers *c* = 3, both the AUC and AUPR of the model reached their optimal values. When the number of layers is 2, the AUC value is 0.9145, which is approximately 3.1% lower than that of three layers. When the number of layers is 4, the AUC value is 0.9273, a decrease of about 1.8%, and when the number of layers is 5, the AUC value is 0.9021, a decrease of approximately 4.4%. This indicates that with a three-layer convolutional network structure, the model can balance feature diversity and computational complexity, achieving the best performance.

In addition, we used Xavier initialization for the model’s parameters and the RMSprop optimizer during training. The learning rate was set to 0.0001, and weight decay was set to 0.0001.

### 2.4. Ablation Experiments

To assess the contribution of each module in our proposed model to the final prediction accuracy and evaluate the impact of each module on the model’s overall performance, we performed a series of ablation studies. The key components of the model worth investigating are the feature extraction for circRNAs and drugs, as well as the combined feature extraction of the two. The two main feature extraction parts are the MSCNN and the AAE.

To extract better features from circRNAs and drugs, one important part of the model is the MSCNN, which is designed to extract multi-scale features from the input data. To assess its contribution, we designed the AAECDAnoMSCN model, which retains the AAE module but omits the MSCNN module. This experiment helps to determine the role of the MSCNN in extracting useful features and enhancing the model’s learning ability.

In the AAE, we designed two additional models: AAECDA_no_encoder and AAECDA_no_discriminator, to separately evaluate the impact of the encoder and discriminator modules on model performance.

1. AAECDA_no_encoder removes the encoder, and the original input data are processed directly by the discriminator without encoding. This is equivalent to using the raw input data for adversarial training without compressed representations.
2. AAECDA_no_discriminator degrades the AAE into a regular autoencoder, where the model structure contains only the encoder and decoder without adversarial training. This design evaluates the importance of the discriminator in guiding the AAE to learn the latent space distribution.

**Figure 6:**
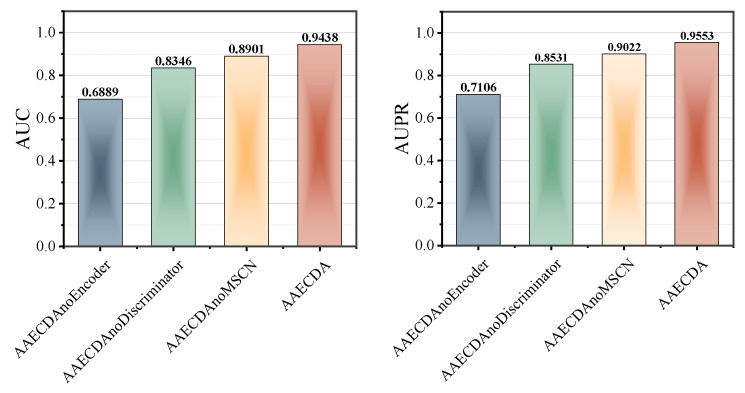
The results of AAECDA and its variants in the ablation study.

As shown in Figure 6, the AUC and AUPR values of AAECDA are significantly higher than those of AAECDA_no_MSCN, AAECDA_no_encoder, and AAECDA_no_discriminator. Table 3 shows that its remaining performance metrics are also always superior to other ablation methods, indicating that each module in the AAECDA model plays an important role in enhancing predictive performance.

Specifically, the MSCNN provides the capability to capture multi-scale features during the feature extraction phase, which is crucial for the model’s accuracy and generalization ability. The ablation experiment results demonstrate that when the MSCNN module is omitted (i.e., in the AAECDA_no_MSCN model), the overall performance of the model significantly decreases, proving the critical role of the MSCNN in effectively extracting complex and multi-scale information from the input data.

In the ablation study of the AAE component, both the AAECDA_no_encoder model, which removes the encoder, and the AAECDA_no_discriminator model, which removes the discriminator, exhibited performance degradation. The AAECDA_no_encoder model shows that without the encoder for data compression, the model’s predictive ability is noticeably weakened, indicating the encoder’s crucial role in reducing data redundancy and focusing on key features. Meanwhile, the AAECDA_no_discriminator model, which lacks the discriminator, experienced even more significant performance degradation, further illustrating the importance of the discriminator in adversarial training. By guiding the encoder to learn the latent space distribution, the discriminator enables the encoder to more effectively capture the structural information and differences in the data.

### 2.5. Performance Under the Blind Test Set

In previous studies, we employed fivefold and tenfold cross-validation to evaluate the performance of the model. Although this approach is effective for training and validating within the dataset, it cannot fully assess the model’s predictive ability in real-world scenarios, where the associations between circRNAs and drugs are usually unknown. To address this issue, we introduced a blind test set to evaluate the model’s performance on truly unseen data.

The blind test set was constructed by randomly sampling 20% of the total samples from the original dataset, ensuring that each sample had an equal probability of being selected. Importantly, the blind test set was completely isolated from the model training and hyperparameter optimization processes. The remaining 80% of the data was used for model selection and hyperparameter tuning through 5-fold cross-validation. After training, we evaluated the model’s performance on the blind test set to assess its generalization ability to new circRNA-drug interactions. To ensure the robustness of our results, we repeated the blind test set evaluation five times with different random splits and reported the average performance metrics.

**Figure 7:**
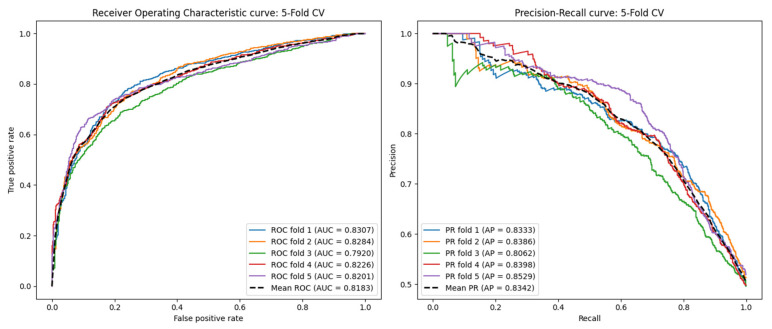
ROC curves and PR curves in fivefold cross-validation under the blind test set.

The ROC and PR curves for the blind test set are shown in Figure 7. Additionally, we compared our model with two state-of-the-art methods, GATECDA and MNGACDA, using the same blind test set division method. The experimental results, presented in Table 4, demonstrate that our model achieved an AUC of 0.8183 on the blind test set, outperforming both GATECDA (AUC = 0.7760) and MNGACDA (AUC = 0.7947). This indicates that our model has a strong generalization ability for predicting circRNA-drug interactions in real-world scenarios.

### 2.6. Case Studies

**Figure 8:**
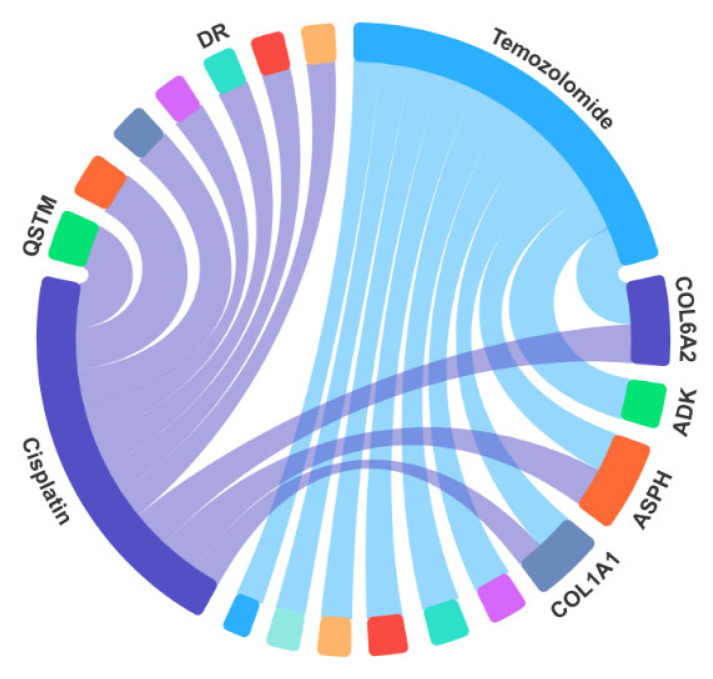
circRNAs association with Temozolomide and Cisplatin.

In our study, we employed the AAECDA-based model. Given that known associations were obtained from the GDSC database [23], we utilized the circRNA-drug sensitivity associations from GDSC as the training set and the circRNA-drug associations from the CTRP database [24] as the test set. To predict 10 potential circRNA-drug association combinations, we selected two specific drugs: Temozolomide and Cisplatin. The predicted scores were ranked from highest to lowest, where higher scores indicate stronger potential associations. These combinations may have therapeutic potential for certain diseases, and some have been supported by existing literature. The associations between circRNAs and drugs can be visually represented using Figure 8.

Temozolomide [25] is an alkylating antitumor drug that is primarily employed in the treatment of malignant brain tumors, including glioblastoma multiforme (GBM) and anaplastic astrocytoma. This oral chemotherapy drug boasts excellent bioavailability and tissue penetration, particularly its ability to cross the blood-brain barrier, making it highly effective in treating brain tumors. It is regarded as one of the standard treatments for gliomas. As indicated in Table 5, of the top 10 circRNAs predicted to be associated with Temozolomide, 8 have already been validated by the CTRP through literature evidence.

Cisplatin [26] is a well-established chemotherapy drug extensively used in clinical settings, classified under platinum-based anticancer agents. This drug is mainly utilized in treating a variety of solid tumors, such as those affecting the head and neck, lungs, bladder, ovaries, and testes. Cisplatin exerts its anticancer effects by binding to DNA and interfering with normal cellular functions. The DNA damage it causes activates several cellular stress responses, including the activation of the *p53 gene*, which leads to apoptosis (programmed cell death). It is considered one of the cornerstone drugs for treating various cancers. As presented in Table 6, 9 of the top 10 predicted circRNAs associated with Cisplatin have been confirmed by the CTRP, with additional support from existing research literature.

## 3. Materials and Methods

### 3.1. Dataset

The datasets and processing methods employed in this study are detailed as follows. We utilized a dataset originally proposed by Deng et al. [9], where circRNA-drug sensitivity associations were sourced from the circRic database [27], and drug sensitivity data were retrieved from the GDSC database [23]. This dataset encompasses 80,076 associations involving 404 circRNAs and 250 drugs. To establish the relationships between each circRNA and drug sensitivity pair, the Wilcoxon test was applied. Correlations with a false discovery rate of less than 0.05 were classified as significant associations. The benchmark dataset used in this study includes only the circRNA-drug sensitivity pairs with significant associations, comprising a total of 4134 associations, 218 drugs, and 271 circRNAs. From this, we constructed the circRNA-drug association matrix $A\in R^{271\times 218}$. For elements in A, $A_{ij}=1$ indicates that circRNA *i* is associated with the sensitivity of drug *j*, otherwise $A_{ij}=0$, where *i* and *j* are the indices of circRNAs and drugs in A, respectively. Furthermore, in order to compute the similarity between circRNAs and drugs, we retrieved the circRNA host gene sequences from the NCBI Gene database [28], while the drug structural information was gathered from the PubChem database [29] provided by NCBI. This dual-source data acquisition allowed for a comprehensive analysis of both genetic and chemical features.

### 3.2. Construction of the Similarity Network

#### 3.2.1. Sequence Similarity of Host Genes of circRNAs

Since circRNAs are formed from exons, introns, or a combination of both from their host genes, their sequence information is closely related to the host gene. If two circRNAs originate from similar or identical genomic regions, their host gene sequences will exhibit higher similarity. Therefore, by comparing the sequences of host genes, potential circRNA similarities can be identified. The similarity of circRNAs can be calculated using the sequence information of their host genes. The Levenshtein distance metric [30] is a tool for measuring the difference between two strings. By calculating the Levenshtein distance between two circRNA sequences, the degree of similarity between them can be quantified. We denote the similarity between circRNAs as $SS_{C}\in R^{271\times 271}$.

(5) $$SS_{C_{\mathrm{leven}}}(c_{i},c_{j})=1-\frac{\mathrm{trans}}{\mathrm{len}(c_{i})+\mathrm{len}(c_{j})}$$

where *trans* represents the minimum cost of transforming one circRNA into another, and len(⋅) denotes the length of the circRNA sequence.

#### 3.2.2. Structural Similarity of Drugs

Obtaining the similarity between drugs by comparing their chemical structures is one of the key methods in drug design and screening. First, after acquiring the chemical structure information of drugs from the PubChem database, RDKit [31] is used to compute the topological fingerprints for each drug. Then, the Tanimoto method is employed to calculate their structural similarity [32]. As a result, we obtain the structural similarity matrix between drugs, denoted as $SS_{D}\in R^{218\times 218}$.

#### 3.2.3. Gaussian Interaction Profile Kernel Similarity of circRNAs and Drugs

In circRNA-drug interaction data, data sparsity is a prevalent challenge. To address this, GIP kernel similarity has been extensively utilized in prior research for similarity calculations [33,34,35]. By leveraging interaction profiles, GIP kernel similarity aids in overcoming the difficulties associated with sparse data, thereby improving the model’s generalization capability. The GIP kernel similarity matrix for circRNA is calculated from the association matrix A, which is denoted as

$GS_{C}\in R^{271\times 271}$, and the calculation is as follows:

(6) $${\mathrm{GS}}_{C}\left(c_{i},c_{j}\right)=\exp\left(-\eta_{c}\left\|\mathrm{IP}\left(c_{i}\right)-\mathrm{IP}{\left(c_{j}\right)}^{2}\right\|\right)$$

where $GS_{C}\in R^{M\times M}$, $IP(c_{i})$ refers to the column corresponding to circRNA $c_{i}$ within the circRNA-drug association matrix, while the parameter $\eta_{c}$ is employed to control the kernel bandwidth. The parameter $\eta_{c}$ is calculated as the average number of their associations. It is defined as follows:

(7) $$\eta_{c}=\eta_{c}^{\prime }/\left(\frac{1}{n_{c}}\sum_{k=1}^{n_{c}}\mathrm{IP}{\left(c_{k}\right)}^{2}\right)$$

where $\eta_{c}=1.0$, and $\eta_{c}$ represents the number of circRNAs. Similarly, the similarity matrix for drugs is denoted as $GS_{D}\in R^{218\times 218}$, and the GIP kernel similarity is calculated as follows:

(8) $${\mathrm{GS}}_{D}\left(d_{i},d_{j}\right)=\exp\left(-\eta_{d}\left\|\mathrm{IP}\left(d_{i}\right)-\mathrm{IP}{\left(d_{j}\right)}^{2}\right\|\right)$$

$IP(d_{j})$ represents the row corresponding to drug $d_{j}$ in the association matrix. The parameter $\eta_{d}$ is the same as above:

(9) $$\eta_{d}=\eta_{d}^{\prime }/\left(\frac{1}{n_{d}}\sum_{k=1}^{n_{d}}\mathrm{IP}{\left(c_{k}\right)}^{2}\right)$$

where $\eta_{d}=1.0$ is the number of drugs.

#### 3.2.4. Similarity Fusion

As previously mentioned, we have separately calculated the similarities between circRNAs and drugs. To obtain more accurate similarities and integrate more biological information, we fuse the circRNA similarities with their Gaussian kernel similarities to construct a circRNA integrated similarity matrix, denoted as $S_{C}$.$S_{C}$ is defined as follows:

(10) $$S_{C_{ij}}=\left\{\begin{array}{c} \frac{\left({SS}_{C_{ij}}+GS_{C_{ij}}\right)}{2},\;\;\mathrm{if}\;SS_{C}\neq 0\\ GS_{C_{ij}},\;\;\;\mathrm{otherwise} \end{array}\right.$$

Similarly, the integrated similarity matrix for drugs is calculated in the same manner as follows:

(11) $$S_{D_{ij}}=\left\{\begin{array}{c} \frac{\left({SS}_{D_{ij}}+GS_{D_{ij}}\right)}{2},\;\;\mathrm{if}\;SS_{D}\neq 0\\ GS_{D_{ij}},\;\;\;\mathrm{otherwise} \end{array}\right.$$

#### 3.2.5. Multi-Scale Convolutional Neural Network

Based on the integrated similarities mentioned above, we input the fused features into a multi-scale convolutional neural network (MSCNN). In a traditional Convolutional Neural Network (CNN), convolutional layers extract features by applying fixed-size filters [36]. However, filters of a single scale may not capture all feature information. For instance, smaller filters are better at capturing detailed features, while larger filters are more suited to capturing global structures. Therefore, we use multiple convolutional kernels of different sizes to capture feature representations at different scales, which helps the model obtain better feature representations.

The core concept of a MSCNN involves utilizing multiple convolutional kernels of varying sizes within the same layer. The outputs from these different kernels are then concatenated to create a multi-scale feature representation. This method offers the advantage of capturing both fine-grained and coarse-grained feature information simultaneously, thereby enhancing the model’s expressiveness and overall performance. Specifically, the network includes three parallel convolutional layers, each using convolutional kernels of sizes 3, 5, and 7 to perform convolution operations on the input features. Each convolutional layer extracts feature representations at different scales, providing richer and more diverse feature representations for subsequent analysis.

From the integrated similarity matrices of circRNAs and drugs, corresponding similarity vectors can be obtained, which serve as their respective feature representations. Each circRNA-drug pair in the dataset is denoted as $\left(c_{i},d_{i}\right)$, where $c_{i}$ represents the similarity vector of each circRNA, and $d_{i}$ represents the similarity vector of each drug. The numbers of circRNAs and drugs in the dataset are denoted as $c_{l}$ and $d_{l}$, respectively. Based on this, we learn better feature representations of vector $c_{i}$ from the feature vectors $E_{c_{i}}$. Specifically, the feature representation generated by the convolution of vector $E_{c_{i}}$ using the l-th filter is

(12) $$s_{c_{i}}^{l}=\psi \left(W_{c_{i}}^{l}*E_{c_{i}}+b_{c_{i}}^{l}\right)$$

where * represents the convolution operation, $W_{c_{i}}^{l}$ is the weight matrix, $b_{c_{i}}^{l}$ is the corresponding bias term, and $s_{c_{i}}^{l}$ denotes the features obtained through the *l* filter. Additionally, ψ(⋅) is the nonlinear activation function. K×n represents the size of the filter. Similarly, the feature representation for drugs is denoted as $s_{d_{i}}^{l}$. Then, the obtained feature vectors are concatenated in the feature merging layer to form the integrated feature representation, which is expressed as:

(13) $$h_{c_{i}}=Concat\left(s_{c_{i}}^{1}\|s_{c_{i}}^{2}\|\ldots \|s_{c_{i}}^{l}\right)$$

where Concat(⋅) represents the concatenation operation for the features. Similarly, the integrated features for drugs are denoted as $h_{d_{i}}$.

Next, the fully connected layer further extracts and compresses the feature information of both through nonlinear transformations. The fully connected layer extracts and compresses the feature information through a nonlinear transformation, and the operation is represented as follows:

(14) $$x_{c_{i}}=\phi \left(\begin{array}{c} W_{c_{i}}x_{c_{i}}+b_{c_{i}} \end{array}\right)$$

where $W_{c_{i}}$ represents the weights of the fully connected layer, $b_{c_{i}}$ is the corresponding bias matrix, and ϕ(⋅) is the activation function. Similarly, the feature of the drug after passing through the fully connected layer is denoted as $x_{d_{i}}$.

Finally, the feature vectors $x_{c_{i}}$ and $x_{d_{i}}$ for circRNAs and drugs processed by the fully connected layers are concatenated to form the final integrated feature representation x, which is expressed as follows:

(15) $$x=\mathrm{Concat}(x_{c_{i}}∥x_{d_{i}})$$

The integrated feature representations of circRNAs and drugs will be input into the subsequent Adversarial Autoencoder (AAE) for more accurate prediction of the associations between diseases and circRNAs.

### 3.3. AAECDA

In this study, we developed a circRNA-drug association prediction model based on an adversarial autoencoder, termed AAECDA. The model is primarily composed of two parts: a MSCNN and an AAE. First, the input to the model is the integrated feature information of circRNA and drugs, which is fed into the MSCNN for feature extraction. The extracted circRNA and drug similarity representations are then input into the AAE for further feature learning. The AAE incorporates a discriminator network, enabling the encoder to not only reconstruct the input features but also generate latent representations in the hidden space that align with the true data distribution. After training, the features output by the encoder are used as input to a deep neural network (DNN) for the final association prediction. The DNN applies multiple layers of nonlinear transformations to further refine and optimize the feature representations, ultimately generating the predicted circRNA-drug association results.

**Figure 9:**
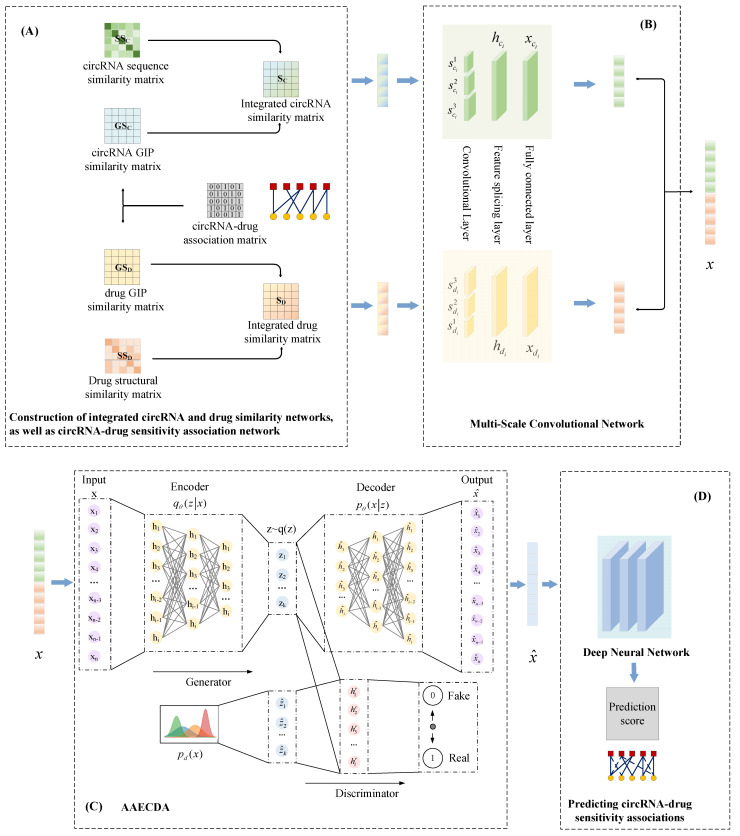
Overall architecture of AAECDA. (**A**) Construction of similarity network, (**B**) Details of extracting integrated features using MSCNN, (**C**) Details of extracting latent representations using AAE, (**D**) DNN predicting association scores.

The proposed circRNA-drug prediction model, AAECDA, is illustrated in Figure 9 and consists of the following steps:

(1) Construction of similarity networks, as well as the sensitivity association network.
(2) Extraction of integrated circRNA and drug features using the MSCNN.
(3) Extraction of the latent representations of circRNA and drugs using the AAE.
(4) Inputting the extracted latent representations into the DNN to predict the circRNA-drug association score.

#### 3.3.1. Adversarial Autoencoder

The Adversarial Autoencoder (AAE) is a deep learning model that integrates the strengths of an Autoencoder (AE) with a Generative Adversarial Network (GAN) [37]. It combines the reconstruction abilities of an autoencoder with the adversarial training of a GAN to effectively learn the latent representation of data. AAE incorporates an adversarial network, typically a discriminator, that aims to align the distribution of the latent space with a predefined prior distribution. Throughout the training, the discriminator and autoencoder are trained simultaneously with opposing objectives. The discriminator’s task is to differentiate between genuine latent variables and those produced by the encoder, while the encoder works to fool the discriminator by making its generated latent variables closely resemble the prior distribution. As a generative autoencoder, AAE employs variational inference and GANs to enforce a specific prior distribution on the feature space. The main benefit of this method lies in its capacity to generate features that align with a predefined prior distribution while capturing the underlying data manifold, irrespective of the process’s state.

A drawback of traditional AE-based feature extraction methods is that the distribution of the extracted features tends to be random and unregulated. To overcome this limitation, we propose a circRNA-drug association prediction method that utilizes the AAE.

A typical AE learns to generate an output identical to the input data x, with the encoder network and decoder network as its two components. The encoder extracts the feature vector z, and the decoder reconstructs the original vector. However, a limitation of AEs is that they randomly map input data into the feature space, resulting in uncontrolled feature distributions. To address this issue, AAE incorporates GAN. The generator (G) and discriminator (D) are the primary components of GANs, which are optimized through adversarial training [38]. The generator’s objective is to learn the data distribution, while the discriminator’s role is to evaluate the similarity between the generated data z and the real data x. Unlike the Variational Autoencoder (VAE), backpropagation through KL divergence requires an explicit functional form of the prior distribution. In contrast, AAEs only require sampling from the prior distribution, which enhances the model’s flexibility.

We use AAE for feature extraction. The integrated feature representation x of circRNAs and drugs mentioned above is supplied to the encoder of the AAE to obtain low-dimensional latent feature representations. Part of the AAE’s encoder is treated as the generator (G) to generate feature vectors analogous to real data, while the discriminator (D) is trained to discern between the distribution of the real data $p_{d}(x)$ and the distribution of the extracted feature vectors q(z). Here, we define the posterior distribution of the hidden layer q(z) as shown below:

(16) $$q(z)=\int_{x}q_{\phi }(z|x)p_{d}(x)dx$$

In the equation, $q_{\phi }(z|x)$ represents the encoder network.

The training process of the AAE model involves two distinct stages: the reconstruction stage and the regularization stage. In the reconstruction phase, the encoder and decoder undergo training with the objective of minimizing the reconstruction error L. The reconstruction error L is specified as follows:

(17) $$L(\phi ,\theta ;x)=-E_{q_{\phi }(z|x)}[\log(p_{\theta }(x|z))]$$

In the equation, $p_{\theta }(x|z)$ represents the decoder network. In the regularization stage, the adversarial network uses the cross-entropy loss function V to train the discriminator to distinguish between real experimental samples and generated samples, and update the parameters of the discriminator *λ*, as shown below:

(18) $$-V(\phi ,\lambda ;x,z)=-\log(d_{\lambda }(p(z)))-\log(1-d_{\lambda }(q(z)))$$

In this equation, p(z) represents the prior distribution and q(z) represents the posterior distribution. Then, Training the generator and updates ϕ as follows:

(19) $$-V(\phi ,\lambda ;x,z)=\log(d_{\lambda }(q(z)))$$

Finally, the reconstruction loss and regularization loss are minimized through backpropagation.

After being decoded by the L-layer decoder, the reconstructed feature representation of circRNA and drugs is denoted as $\hat{x}$. Specifically, the generation process of $\hat{x}$ is as follows:

(20) $$\hat{x}=p_{\theta }(\hat{x}|z)$$

Through adversarial training, AAE approximates the distribution of the latent space to a predefined prior distribution (such as a standard normal distribution), making the latent space representation more regular and meaningful. This approach enhances the interpretability of the latent space and makes generating new samples from the latent space more stable. Compared to VAE, AAE does not require an exact specification of the prior distribution’s functional form. Instead, adversarial training ensures that the latent distribution approximates a target distribution, increasing the model’s flexibility and adaptability. Through adversarial training, AAE is able to learn more stable and generalized latent representations, which helps improve the model’s performance when handling new data or performing transfer learning.

#### 3.3.2. Association Prediction Based on Deep Neural Networks

After obtaining the latent feature representations using the previously described AAE, we employ the DNN to predict the associations between circRNAs and drugs. Each layer typically contains several neurons that are interconnected with the neurons in both the preceding and subsequent layers, forming a complex network structure. DNNs offer significant advantages, particularly in their ability to manage complex nonlinear relationships. By utilizing multiple hidden layers and nonlinear activation functions, DNNs are capable of capturing intricate patterns and features embedded within the data. Additionally, DNN models trained on large datasets exhibit strong generalization capabilities, performing well on previously unseen data and minimizing the risk of overfitting.

First, $\hat{x}$ is input into the first fully connected layer to generate the hidden layer representation $m_{1}$. Then, the hidden layer representation undergoes multiple transformations through fully connected layers, resulting in the final high-level feature representation $m_{n}$. After each fully connected layer, we introduce Dropout and Batch Normalization operations to prevent overfitting and accelerate model convergence.

Finally, the last hidden layer representation $m_{n}$ is input into the output layer. The output layer passes through the Sigmoid activation function to generate the final predicted probability *p*:

(21) $$p=\mathrm{Sigmoid}(W_{\mathrm{out}}\cdot m_{n}+b_{\mathrm{out}})$$

The Sigmoid activation function restricts the output value between [0, 1], representing the predicted association probability.

For the training of the DNN, since our task is a binary classification problem of predicting circRNA-drug associations, we use the binary cross–entropy loss function. The formula for binary cross—entropy loss is:

(22) $$L=-\frac{1}{N}\sum_{i=1}^{N}[y_{i}\log(p_{i})+(1-y_{i})\log(1-p_{i})]$$

where N is the number of samples, $y_{i}$ is the true label of the i-th sample (taking values 0 or 1), and $p_{i}$ is the predicted probability of the *i*-th sample. This loss function measures the difference between the predicted probabilities and the actual labels. Minimizing the binary cross—entropy loss during the training process helps the DNN to make more accurate predictions.

In our code, we set the threshold for classification as 0.5. That is, when p≥0.5, the prediction result is positive (association exists); when p<0.5, the prediction result is negative (no association exists).

Once the model has been trained, it can be utilized to identify and predict new associations between previously unknown circRNA and drug pairs within the dataset.

## 4. Conclusions

With the advancement of research on cancer and other diseases, increasing evidence shows that the expression of circRNAs in human cells can impact drug sensitivity, thereby playing a crucial role in treatment outcomes. As a result, predicting the associations between circRNAs and drug sensitivity not only facilitates the development of novel therapeutics but also aids in overcoming drug resistance in cells, ultimately improving treatment efficacy. There is an urgent need to develop a computational method for identifying potential associations. In this study, we introduce a novel computational framework, AAECDA. We first built a bimodal network based on known association data and quantified the similarities between drugs and circRNAs.

Next, we processed the network using a multi-scale convolutional neural network and further extracted and refined features through an adversarial autoencoder. Finally, DNN is used to learn low-dimensional features for prediction.

To assess the effectiveness of the AAECDA model, we conducted cross-validation across multiple datasets and compared its performance with various existing methods. The results indicate that AAECDA consistently outperformed other approaches in predicting circRNA-drug associations. In addition, we conducted a case study, and the results further validated the effectiveness of the AAECDA model in predicting novel associations. However, the experimentally validated associations available are still limited, which may affect the accuracy of the model’s predictions to some extent. Moving forward, we aim to gather more circRNA-drug sensitivity data, along with additional bioinformatics data such as drug-disease and circRNA-disease associations, to improve the model’s predictive performance by integrating multi-source data.

In conclusion, while current methods for predicting associations between drug sensitivity and circRNAs have certain limitations, this study offers valuable insights for advancing research and applications in this area. Future work will be necessary to enhance the accuracy and reliability of prediction models, paving the way for more effective approaches in this field.

## Figures and Tables

**Table 1:** Comparison with other methods based on 5-CV.

	GATECDA	MNGACDA	MKGCN	LAGCN	AAECDA
AUC	0.8846	0.9139	0.8664	0.8642	0.9438
AUPR	0.8928	0.9209	0.8662	0.8738	0.9553
F1_SCORE	0.8279	0.8489	0.8023	0.8084	0.8305
ACCURACY	0.8190	0.8310	0.7985	0.8019	0.8480
RECALL	0.8348	0.8498	0.8186	0.8265	0.8534
SPECIFICITY	0.8065	0.8343	0.7889	0.7539	0.8785
PRECISION	0.8273	0.8590	0.7857	0.7912	0.9302

**Table 2:** Comparison with other methods based on 10-CV.

	GATECDA	MNGACDA	MKGCN	LAGCN	AAECDA
AUC	0.8918	0.9182	0.8683	0.8710	0.9464
AUPR	0.9015	0.9249	0.8769	0.8818	0.9570
F1_SCORE	0.8267	0.8373	0.8047	0.8133	0.8410
ACCURACY	0.8271	0.8427	0.8026	0.8076	0.8407
RECALL	0.8312	0.8536	0.8173	0.8312	0.8437
SPECIFICITY	0.8135	0.8323	0.7973	0.7486	0.8881
PRECISION	0.8225	0.8517	0.7937	0.7956	0.9383

**Table 3:** Results of ablation experiments for AAECDA.

Variant	AUC	AUPR	F1_SCORE	ACCURACY	RECALL	SPECIFICITY	PRECISION
AAECDAnoEncoder	0.6889	0.7106	0.7205	0.7022	0.7864	0.6975	0.7764
AAECDAnoDiscriminator	0.8346	0.8531	0.8014	0.8012	0.8248	0.7967	0.8083
AAECDAnoMSCN	0.8901	0.9022	0.8095	0.8237	0.8533	0.7991	0.8238
AAECDA	0.9438	0.9553	0.8305	0.8480	0.8534	0.8785	0.9302

**Table 4:** Comparison with other methods on the blind test set based on 5-CV.

	GATECDA	MNGACDA	AAECDA
AUC	0.7760	0.7947	0.8183
AUPR	0.7821	0.8013	0.8342

**Table 5:** The Top 10 circRNAs associated with the drug Temozolomide.

Drug	Rank	circRNA	Evidence
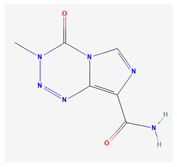 Temozolomide	1	COL6A2	CTPR
	2	ADK	CTPR
	3	ASPH	CTPR
	4	COL1A1	CTPR
	5	EFEMP1	CTPR
	6	RPN1	NA
	7	MYH9	CTPR
	8	ADGRG1	CTPR
	9	COPG1	CTPR
	10	KATNB1	NA

**Table 6:** The Top 10 circRNAs associated with the drug Cisplatin.

**Drug**	**Rank**	**circRNA**	**Evidence**
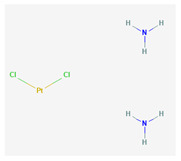 Cisplatin	1	SQSTM1	CTPR
	2	CALR	CTPR
	3	ASPH	CTPR
	4	COL6A2	CTPR
	5	LTBP1	CTPR
	6	VIM	CTPR
	7	WDR5	CTPR
	8	MYADM	CTPR
	9	POLR2A	NA
	10	COL1A1	CTPR

## Data Availability

The required data are available on GitHub https://github.com/yjslzx/GATECDA (accessed on 10 June 2024).
